# A residue management machine for chopping paddy residues in combine harvested paddy field

**DOI:** 10.1038/s41598-023-32148-9

**Published:** 2023-03-28

**Authors:** Chelpuri Ramulu, Raj Narayan Pateriya, Mude Arjun Naik, Dinesh Kumar Vishwakarma, Alban Kuriqi, Nadhir Al-Ansari, Abed Alataway, Ahmed Z. Dewidar, Mohamed A. Mattar

**Affiliations:** 1 Department of Farm Machinery and Power Engineering, G.B. Pant University of Agriculture and Technology, Pantnagar, Uttarakhand 263145 India; 2 Dr. Rajendra Prasad Central Agricultural University Pusa, Samastipur, Bihar 848125 India; 3 Department of Irrigation and Drainage Engineering, G. B. Pant University of Agriculture and Technology, Pantnagar, Uttarakhand 263145 India; 4 CERIS, Instituto Superior T´Ecnico, University of Lisbon, 1649–004 Lisbon, Portugal; 5 Civil Engineering Department, University for Business and Technology, Pristina, Kosovo; 6 Department of Civil, Environmental, and Natural Resources Engineering, Lulea University of Technology, 97187 Lulea, Sweden; 7 Prince Sultan Bin Abdulaziz International Prize for Water Chair, Prince Sultan Institute for Environmental, Water and Desert Research, King Saud University, Riyadh, 11451 Saudi Arabia; 8 Department of Agricultural Engineering, College of Food and Agriculture Sciences, King Saud University, Riyadh, 11451 Saudi Arabia; 9 Agricultural Engineering Research Institute (AEnRI), Agricultural Research Centre, Giza, 12618 Egypt

**Keywords:** Mechanical engineering, Sustainability

## Abstract

Nowadays, Combine Harvesters are the most commonly used device for harvesting crops; as a result, a large amount of plant material and crop residue is concentrated into a narrow band of plant material that exits the combine, challenging the residue management task. This paper aims to develop a crop residue management machine that can chop paddy residues and mix them with the soil of the combined harvested paddy field. For this purpose, two important units are attached to the developed machine: the chopping and incorporation units. The tractor operates this machine as the main source, with a power range of about 55.95 kW. The four independent parameters selected for the study were rotary speed (R_1_ = 900 & R_2_ = 1100 rpm), forward speed (F_1_ = 2.1 & F_2_ = 3.0 Kmph), horizontal adjustment (H_1_ = 550 & H_2_ = 650 mm), and vertical adjustment (V_1_ = 100 & V_2_ = 200 mm) between the straw chopper shaft and rotavator shaft and its effect was found on incorporation efficiency, shredding efficiency, and trash size reduction of chopped paddy residues. The incorporation of residue and shredding efficiency was highest at V_1_H_2_F_1_R_2_ (95.31%) and V_1_H_2_F_1_R_2_ (61.92%) arrangements. The trash reduction of chopped paddy residue was recorded maximum at V_1_H_2_F_2_R_2_ (40.58%). Therefore, this study concludes that the developed residue management machine with some modifications in power transmission can be suggested to the farmers to overcome the paddy residue issue in combined harvested paddy fields.

## Introduction

Crop harvesting has produced straw as a by-product for centuries, making it an important agricultural resource^1^. Despite its natural resources, straw after harvest is of immense value as a soil fertility and structure enhancer^2^. The second principle of conservation agriculture is the retention of residues, which contributes to the health of the soil, reduces soil erosion, and improves moisture content in the soil, improving soil organic content, thus increasing crop yields and the use of energy^3^. Various factors influence field residue retention, including crop, soil, climate, slope, and residue management practices.

There was a tremendous increase in yields of major crops from 1949–50 to 2017–18. It was about 379.8% in paddy, 1460.4% in wheat, 1337.6% in maize, 209.9% in pulses, 388.7% in oilseeds, and 669.3% in sugarcane^4^**.** Most crop residues are burned in fields instead of used to feed animals, make nutritional compost, or plant mushrooms, even though they can be sparked into bio-energy for rural supply and development^5,6^. There is a direct correspondence between crop productivity and power availability in agriculture production. Approximately 371 million tons of crop residue are produced in India each year, of which wheat residues represent 27–36%, and paddy residues represent 51–57%^7,8^. In the northwest (NW) region of India, namely Punjab, Haryana, and Uttar Pradesh, the residues of paddy crops are burned in situ, which is another common management practice. Burning residues contribute up to 20% of the agricultural waste burning emissions budget in NW India^9^. Each tonne of straw (rice and wheat) on burning releases 3 kg of a particular matter, 60 kg of CO, 1460 kg of CO_2_, 199 kg of ash, and 2 kg of SO_2_^10^_,_ causing significant global warming and acid rain. And about 32–67% of the strawweight and 27–73% of nitrogen are lost due to burning^1,11^. Paddy and wheat straw burning in India in 2000 are predicted to emit 110, 2306, 2, and 84 gigagrams (Gg) of CH_4_, CO, and NOx, respectively^12^. Thus, the country must enhance farm mechanization for food production and quality of life. In many agricultural practices, production costs are quite high due to the intensity of labor that goes into different aspects of farm operations. In contrast, the share of mechanical and electrical power sources increased from 7% to approximately 90% during the same period. In Indian agriculture, there is a preponderance of small operational holdings mainly employed for agriculture operations; this means that the consolidation of land holdings will be necessary to reap the benefits of agricultural mechanization^13^.

Regarding global economic growth, it can be said that agricultural production plays an important role in driving that growth. Undoubtedly, India is the world's second-largest producer of rice and wheat. According to available literature, about 6 tons of residue are produced for every 4 tons of wheat or rice production, representing a huge amount of straw available for safe and appropriate disposal yearly. In a combined harvested field, the farm's total yield of paddy residue will be approximately 12.5 tons per hectare. In comparison, the yield of standing stubbles and loose straws will be approximately 7 tons per hectare and 5.5 tons per hectare, respectively^14^**.** In India, an average of 500 MT per annum of crop residue was produced from different crop species; the major residue obtained from rice and wheat was about 34% and 22%, respectively**.** Out of total crop residue, 360 MT is used for animal feeding, soil mulching, bio-manure, thatching for rural houses, and fuel for domestic and industrial use. After this collection use, there is a surplus amount of residue of 140 MT, out of which 92 MT is burned by farmers every year due to the unavailability of appropriate machines for paddy residue management and mechanized farming linked with low-skilled farm labour^15–18^.

Burning crop residues releases a substantial amount of smoke and soot into the air, causing increased pollution. This process leads to the release of greenhouse gases (GHGs), like carbon dioxide, methane, and nitrous oxide, contributing to global warming. Additionally, it results in the loss of important plant nutrients such as N, P, and K, which negatively impacts the soil properties and a loss of valuable organic carbon and energy-rich residues to the environment. Burning paddy straw on fields releases pollutants into the atmosphere, further aggravating the impact of climate change issues^19^.

In addition to burning crop residues, there are many possible alternatives to the burning of crop residues, including decomposing the residue with a chemical adjuvant, shredding the residue, and then incorporating it into the soil. There are several benefits to shredding trash: it reduces the size of the particles, and therefore more microbes will be able to degrade the residue quickly, increasing the amount of carbon and nitrogen mineralized from the residue. Furthermore, soil incorporation with residues would increase the surface area of residue in contact with soil microbes, speeding up the decomposition process. Crop residue management systems may benefit from straw incorporation to enhance agricultural productivity and sustainability. This is considered the most effective practice for improving soil properties and fertility^20,21^.

It has been recommended that the incorporation of straw 15–20 days before wheat sowing in northwestern India should be a substitute for paddy straw burning^22,23^. Incorporating harvest residue into the soil would also increase the amount of residue surface that would come into contact with microbes living in the soil and, as a result, would speed up the decomposition of the residue. Thus, it is important to emphasize the importance of properly managing crop residues to maintain soil fertility to sustain the high crop productivity achieved in recent years^24–26^. Likewise, the incorporation of crop residues enhanced soil quality in terms of enhanced soil organic carbon, hydraulic conductivity, infiltration rate, water holding, cation exchange capacity, enzymatic activity, and aggregate stability. In general, shredding crop residue has caused a faster degradation rate, as indicated by an increase in carbon and nitrogen mineralization, influenced the peak respiration time, or had no effect on respiration. Multiple tillage operations (2–3 times harrow/power tiller, or rotavator and planker) are needed to incorporate straw and prepare the seedbed for wheat sowing, which raises the cost of cultivation and delays wheat sowing. The straw incorporation in the field is highly time-consuming and requires 6–7 operations. Therefore, farmers opt for burning paddy straw to quickly clean the field for sowing the next wheat crop as it has limited use^27^. The machinery for crop residue management combines a rotavator and straw chopper, a happy seeder, a zero seed drill, a straw baler, and a super straw management system on a combined harvester**.**

Roto till drill was developed for sowing wheat after paddy harvesting^28^. The machine was a rotavator combined with a seed drill. They stated that the machine performance was unsatisfactory in combined harvested paddy fields. Additionally, they claimed that when used after straw had been chopped using a straw chopper, the machine's performance was acceptable^28,29^. A happy combo seeder was developed; it sows the field in one operation while cutting, lifting, and throwing the loose straw and standing stubble. It requires the tractor with 45 hp as a power source and driven by PTO of the tractor with a field capacity of 0.3–0.04 ha/h while operated with a tractor. The satisfactory performance of the machine was found when the straw load was less than 7 t/ha. The build-up of mud and straw on happy seeder tynes was observed in wet clay soils.

In a single operation, the machine harvests the stubbles left after combing, chopped into pieces, and spreads them on the ground^27^. Using a single rotavator or disc harrow operation, the chopped and spread stubbles were easily buried in the soil and decayed after irrigation. Rice straw chopper-cum-spreader works satisfactorily in both loose and stand stubble conditions. However, the chopped straw and stubble required more decomposing time, delaying the next crop's sowing. Clogging of loose straw was observed in the stubble harvester cum chopper. A straw bruiser and a cumber spreader are accessories for the current combine. It was fixed to the rear hood of the combine harvester, behind the chaff sieves, and just in front of the straw walkers. The developed machine's primary purpose was to break up the straw and chaff from the combine's straw walker and sieve into smaller pieces before scattering them back over the harvested field^30^.

Plant residue choppers can be used on various fields, including maize, wheat, rice, cotton, and sugarcane. They are available from manufacturers such as 1JH (China), Tornado (Italy), Croplogix (USA), and RM (France). Two-wheel, four-wheel, and combine harvesters can operate the machines, with the four-wheel tractor version being the most popular. Happy seeder, incorporator, zero seed drill, straw baler, paddy straw chopper, and super straw management system on a combine harvester are commercially available agricultural machinery for crop residue management. However, paddy residue management, which includes cutting, chopping, and incorporation, cannot be accomplished alone by existing machinery. Therefore, there is a need to develop an appropriate residue management machine to completely manage paddy residue of combined harvested paddy fields in a single pass^31^.

This paper aims to develop a machine and evaluate its performance for paddy residue management. The primary goal of the straw chopper cum incorporator is to uniformly reduce the size of residues and incorporate them into the soil in one pass to increase soil fertility and biological activity. It can retain soil moisture and increase soil porosity and aeration for enhanced germination and growth for the next Rabi crop. The proposed residue management machine can cut the stubbles and mix them with soil. Therefore, those facts were considered while developing the present paddy residue management machine.

## Methods

### Design of the developed residue management machine components

A straw chopper and incorporator were combined with an adjustable frame to develop the straw chopper cum incorporator. The prior design consideration for the development of the straw chopper cum incorporator includes the design of its width, which was kept constant at 2100 mm as it is the regular size of implements used by farmers, especially in the northwestern region of India. The recommended rotary speed of the incorporator was kept at 180–210 RPM for proper pulverization of soil during the incorporation of the paddy residue ^32^. Past researchers showed that the flail-type straw chopper performance was best at the rotary speed ranges between 750 and 1900 rpm. The horizontal and vertical adjustment of the straw chopper cum incorporator depends upon the paddy stubble's length after harvesting the crop. The average length of paddy stubble was about 350–600 mm. The horizontal space between the center of the straw chopper rotor axis and the incorporator rotor axis was 550 mm and 650 mm. The vertical space between the center of the straw chopper rotor axis and to incorporator rotor axis was 100 mm and 200 mm^33^. The shape of incorporator blades for paddy residue incorporation was generally used L-type. Therefore, the L-type blades were considered for this study ^32^. Like in the past, the J-type flail blades were used in a flail-type chopper, but those blades did not work satisfactorily in shredding and chopping paddy residue. Inverted gamma-type blades work more satisfactorily than J-type and cutter bar serrated-type blade ^34^. Therefore, inverted gamma-type blades were used in the straw chopper. As per past studies, it is recommended that the rotational speed of the incorporator for proper incorporation of paddy residue was found to be 180–210 rpm^32^.

#### Design of the frame

**Figure 1:**
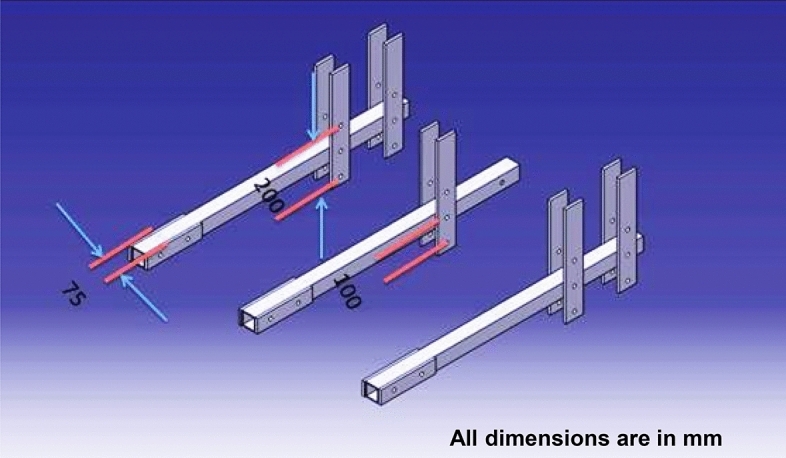
Isometric view of frame used in a developed machine.

The frame was developed using MS (mild steel) square pipes and MS flat. Three square pipes of size 75 × 75 mm of length 800 mm was used for this purpose. The MS flat of size 100 × 25 mm was used for the frame. The developed frame is superior in that either side can be used to adjust the horizontal and vertical clearance between the incorporator shaft and straw chopper rotor shaft. Because the horizontal and vertical clearances may impact the performance, this adjustment was made to enhance the machine's performance ^33^. The frame details are illustrated in Fig. 1.

#### Torque requirement of the developed machine

Calculating the torque requirements of the straw chopper and incorporator separately determined the torque requirements of the straw chopper and incorporator. The sum of the straw chopper and incorporator toque requirement was the total torque requirement of the developed machine. The power requirement was assumed as per recommendations from manufacturers and previous studies. The torque is calculated by using the following equation:

1 $$P = \frac{2 \times \pi \times N \times T}{{60}}$$

where, *P* is the power requirement of the straw chopper, kW, *T* is the torque requirement of the straw chopper, N-m, and *N* is the speed of the rotor shaft of the straw chopper, rpm.

Table 1 provides the torque requirement for the straw chopper cum incorporator. The total torque requirement for the developed machine is 1790.94 N-m, the sum of the individual torque requirement of the straw chopper (435.24 N-m) and incorporator (1355.70 N-m).

**Table 1:** Torque requirement of straw chopper cum incorporator.

Particulars	Power (kW)	Speed (rpm)	Torque, (N-m)
Straw chopper	41	900	435.24
Incorporator	30	210	1355.70
Torque requirement of developed machine	1790.94		

#### Design of belt and pulley

The open belt and pulley system were used for power transmission of the straw chopper cum incorporator. A V-type B-category belt was used in the power transmission system of the developed machine. The length of the belt was determined by using the following equation:

2 $$L = \frac{\pi }{2}\left( {D + d} \right) + 2X + \frac{{\left( {D + d} \right)^{2} }}{4X}$$

where, *L* is the length of the belt (mm),* D* is the diameter of the driven pulley, mm,* d* is the diameter of a driver pulley, mm, and *X* is the Central distance between the driven and driver pulley, mm. The calculated length of the belt of L_1_, L_2,_ and L_3_ are given in Table 2.

**Table 2:** Length of belt calculation table (all units are in mm).

	Diameter of driver pulley (D)	Diameter of driven pulley (d)	Center distance (X)	Length of belt
L1	228.6	165.1	388.1	1397
L2	228.6	420.1	562.1	2159
L3	101.6	228.6	447.4	1422.4

The selection of the number of belts or pulley grooves was done by the following assumptions^23,25,33,35^ as a material of belt mass density of rubber (ρ) is1140 kg/m^3^, permissible stress in rubber belt $\left({\varvec{\upsigma}}\right)$ is 21 MPa, the coefficient of friction between belt and pulley (µ) is 0.30, and the weight of the rubber belt per meter (m) is 4.31 N.

The number of required belts was calculated for the straw chopper and incorporator using the following equations:

3 $$n = \frac{a}{A}$$

where, *n* is the number of belts,* a* is the calculated cross-sectional area of the belt, mm^2^, and *A* is the standard cross-sectional area of the belt, mm^2^. The calculated cross-sectional area (*a*) is obtained by:

4 $$P\left( {s/r} \right) = \left( {\sigma a - mv^{2} } \right) \times v\left( {1 - \frac{1}{{e^{\mu \theta } }}} \right)$$

where, *θ* is the angle of contact, radians, and *v* is the belt's velocity, m/s. The standard cross-sectional area of the belt (A) = bt, where b is the width of the belt in mm, and t is the thickness of the belt in mm. For the B belt type, width and thickness are 17 and 11 mm, respectively^35^.

#### Selection of number of belts for straw chopper

Power assumed for straw chopper *P*_*s*_ = 41 kW; *α* = Angle of contact, degree, therefore,

5 $${\text{Velocity of belt}} = \frac{{{\pi D}_{1} {\text{N}}_{1} }}{60}{ } = 7.77{\text{ m}}/{\text{s}}$$

$$\uptheta =180-2\mathrm{\alpha };\mathrm{sin \, \alpha }= \frac{{\mathrm{r}}_{1}-{\mathrm{r}}_{2}}{\mathrm{x}};\mathrm{ \alpha }=9.59 \, \mathrm{ and \, \theta }=2.80 \, \mathrm{ radians}$$

Therefore, the number of belts required = $\frac{a}{A}$ ; the Number of belts required is 3.

#### Selection of the number of belts required for the incorporator

Power assumed for straw chopper P_r_ = 30 kW

6 $${\text{Velocity of belt}} = \frac{{{\pi D}_{4} {\text{N}}_{4} }}{60}{ } = 2.49{\text{ m}}/{\text{s}}$$

$$\uptheta =180-2\mathrm{\alpha };\mathrm{sin\alpha }= \frac{{\mathrm{r}}_{4}-{\mathrm{r}}_{5}}{\mathrm{x}};\mathrm{ \alpha }=1.84\mathrm{ and \theta }=3.07\mathrm{ radians}$$

Therefore, the number of belts required = $\frac{a}{A}$, Number of belts required is 3.

#### Design of shafts for straw chopper and incorporator

The straw chopper and incorporator shaft diameter were determined using the torsion equation assuming allowable shear stress, $\uptau =70\mathrm{ MPa}$.

7 $$T = \frac{{\uppi }}{16} \times {\uptau } \times d^{3}$$

where, *T* is the twisting moment or torque acting upon the shaft, N-m, ${\varvec{\tau}}$ is the torsional shear stress, MPa, and *d* is the shaft diameter, mm. The diameters of shaft-1, shaft-2, and shaft-3 were calculated using Eq. 7 in Table 3.

**Table 3:** Diameter of both straw chopper and incorporator shafts.

	Rotary speed, rpm	Torque, N-m	Allowable stress, MPa	Diameter of Shaft, mm
Shaft 1, mm	650	602.34	70	≈ 40
Shaft 2, mm	900	435.24	70	≈ 40
Shaft 3, mm	450	633.22	70	≈ 40
Shaft 3, mm	210	1357.59	70	≈ 50

#### Power transmission

**Figure 2:**
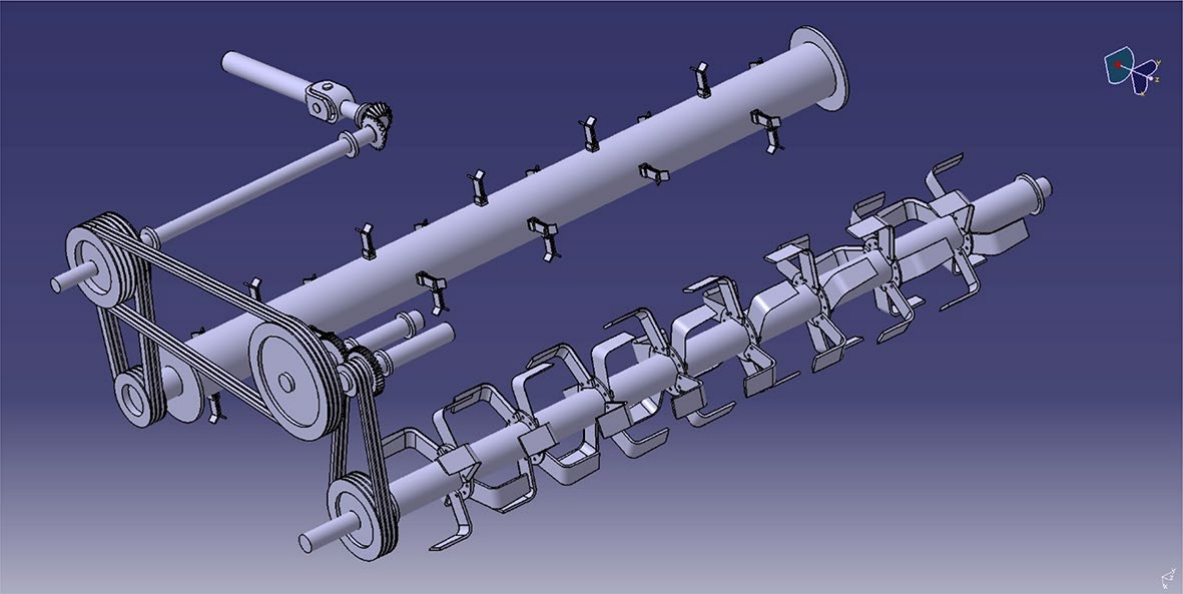
Isometric view of power transmission of a chopper cum incorporator machine.

**Figure 3:**
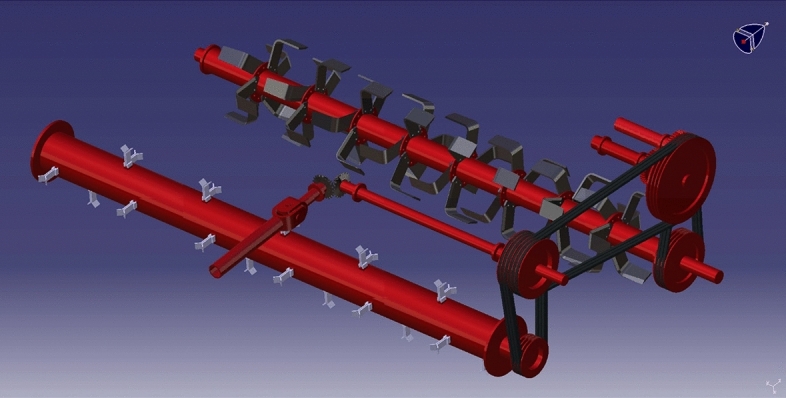
Isometric view of power transmission of a chopper cum incorporator machine.

The belt and pulley system transmitted power from one shaft to another. The belt, pulley, gears, shaft, and bearings were the power transmission components. Various diameters of 5 B-type multi-groove pulleys were used, out of which two pulleys were used at straw chopping machine, *i.e.,* pulley number 1 and pulley number 2, and the remaining three pulleys were used at incorporating machine, *i.e.,* pulley 3, 4 and 5. One set of spur gears (75 teeth) of the same diameter was used; the main purpose of these two gears was to reverse the direction of the rotation pulley 4. The power for pulley four was transmitted from pulley 3. The power transmission system components are described in Figs. 2 and 3.

The power from the PTO (Power take-off) shaft of the tractor was transmitted to the straw chopper gearbox utilizing a telescopic shaft; then, power was transmitted to the rotor shaft of the chopper utilizing a belt and pulley arrangement. An open belt pulley system connected the chopper shaft to the gearbox by a suitable pulley arrangement at its end. Pulley number 1 was placed at the top side, and pulley number 2 on the rotor shaft of the straw chopper. The straw chopper rotor shaft rotates in a clockwise direction. Pulley number 2 has six grooves connected to pulleys 1 and 3. Pulley number 1 receives power from pulley number 2, and pulley 3 receives power from the remaining three grooves of pulley number 2. These entire three pulleys’ have a clockwise direction of rotation. The power from pulley three was mounted on shaft number 1 to shaft number 2, transferred by spur gears. Pulley number 4 was mounted on the respective shaft. The direction of rotation of pulley number 4 was changed to the anticlockwise direction. The power from pulley number 4 was transferred to pulley number 5. Pulley number 5 rotates the rotor shaft incorporator. Therefore, the rotor shaft of the incorporator rotates in the anticlockwise direction.

#### Calculation of velocity ratio

The driver's and driven pulley's speeds are called the velocity ratio. The velocity ratio was calculated by using the following equation:

8 $${\text{Velocity ratio }}\sim { }\frac{{\text{n}}}{{\text{N}}}{ } = { }\frac{{\text{D}}}{{\text{ d}}}$$

Let N, D = speed (rpm) and diameter of a driving pulley (mm), respectively; n, d = speed (rpm) and diameter of driven pulley (mm), respectively; the speed at pulley 1 (N_1_) = 650 rpm, the diameter of pulley 1 (D_1_) = 228.6 mm; the speed at pulley 2 (N_2_) = 900 rpm, the diameter of pulley 2 (D_2_) = 165.1 mm;

9 $${\text{Velocity ratio at the chopper }}\left( {{\text{G}}_{1} } \right) = { }\frac{{{\text{N}}_{2} { } \times {\text{ D}}_{2} }}{{{\text{N}}_{1} { } \times {\text{ D}}_{1} }} = 1.4$$

Speed at pulley 3 (N_3_) = 450, diameter of pulley 3 (D_3_) = 304.8 mm, speed at pulley 4 (N_4_) = 450, diameter of pulley 4 (D_4_) = 101.6 mm, speed at pulley 5 (N_5_) = 200 rpm, diameter of pulley 5 (D_5_) = 228.6 mm:

10 $${\text{Velocity ratio at incorporator }}\left( {{\text{G}}_{2} } \right) = { }\frac{{{\text{N}}_{1} { } \times {\text{ D}}_{1} \times {\text{ D}}_{4} }}{{{\text{N}}_{5} { } \times {\text{ D}}_{3} \times {\text{ D}}_{5} }} = 1.08$$

Main velocity ratio G = G_1_ X G_2_ = 1.51.

#### The calculation for the horizontal and vertical adjustment

The horizontal & vertical clearance between both axes of the straw chopper and incorporator was calculated by using the following equations. Vertical clearance is given by:

11 $${\text{V}} = { }\left( {{\text{R}}_{1} - {\text{R}}_{2} } \right) + \left( {{\text{h}}_{1} - {\text{h}}_{2} } \right)$$

Horizontal clearance is given by:

12 $${\text{H}} = { }H_{2} + \sqrt {\left( {R_{2}^{2} - \left( {R_{2}^{2} - h_{2} } \right)^{2} } \right)}$$

where, *V* is the vertical clearance in mm, *H* is the horizontal clearance, *h*_*1*_ is the length of chopping (= 80 to 100 mm), *R*_*1*_ is the rolling radius of the straw chopper shaft with inverted gamma type of blades (= 170 mm), *R*_*2*_ is the rolling radius of the incorporator shaft with L type blades (= 150 mm) and* h*_*2*_ is the depth of incorporation (= 100 mm). *H*_*2*_ = *1.1 H*_*1*_*.*

13 $$H_{1} = \frac{{R_{1} \sin \alpha + h_{1} + R_{1} \left( {1 + \cos \alpha } \right)}}{\tan \alpha }$$

where, α is the included angle between the ground and the absolute velocity of the chopped paddy residue (= 45°). The vertical clearance and clearances were calculated at about 220 mm and 640 mm, respectively.

#### The developed machine

**Figure 4:**
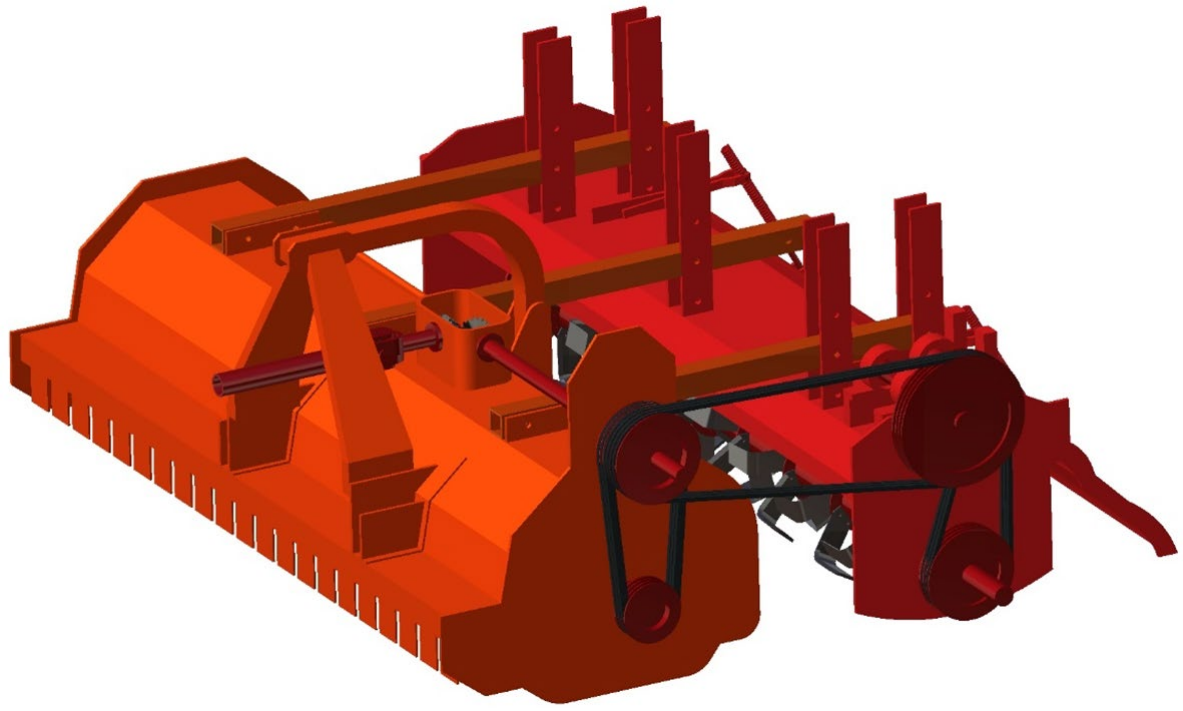
Isometric view of the developed residue management machine.

**Figure 5:**
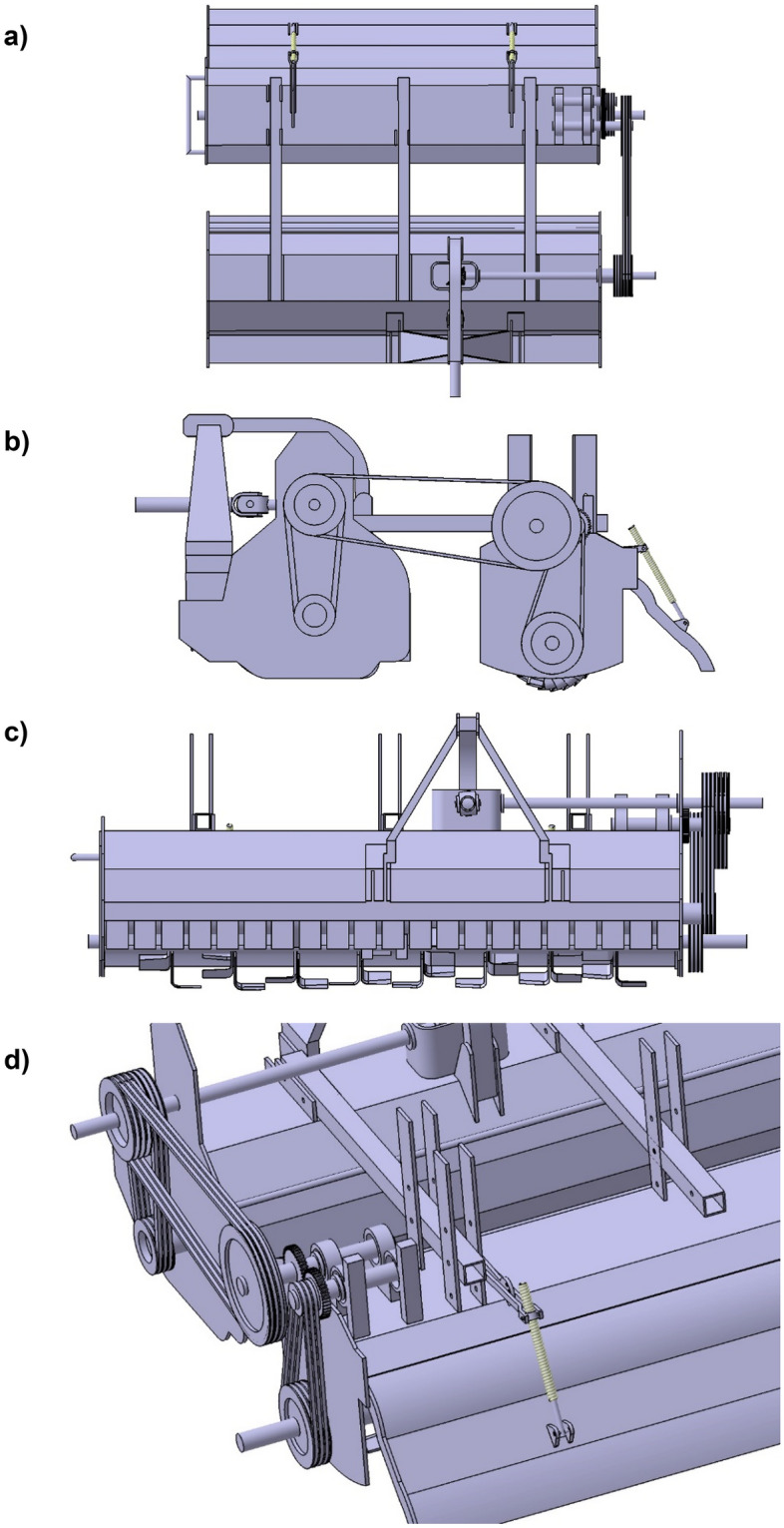
(**a**) Top (**b**) side, (**c**) front, and (**d**) gear views of the developed residue management machine.

The developed machine combined the adjustable frame, the incorporator, and the straw chopper. The straw chopper has inverted gamma-type blades, and processed paddy residues will be incorporated using an incorporator with L-type blades. The developed machine completes cutting, chopping, and incorporating straw in a single pass. Soil health would be improved by increasing the paddy residue decomposition rate and incorporating it into the soil. Figures 4 and 5 depict the developed machine in isometric, top, side, front, and rear views and an isometric view from the rear side. The detailed specification of the developed machine is presented in Table 4.

**Table 4:** Specification of the developed residue management machine.

	Particulars	Specifications
1	Length × Width × Height	1900 × 2100 × 950 mm
2	Weight of the machine (W)	900 kg
3	Power requirement	56 kW
4	Number of working elements in the chopper	22
5	Type of flails in the chopper	Inverted gamma type
6	Speed of Flail shaft in the chopper	900–1500 rpm
7	The shape of working elements in the incorporator	L shape
8	Speed of incorporator shaft	180–210 rpm
9	Number of working elements in incorporator	48
10	Mode of Power Transmission	Belt, pulley, and spur gears

### Performance evaluation of developed machine

**Figure 6:**
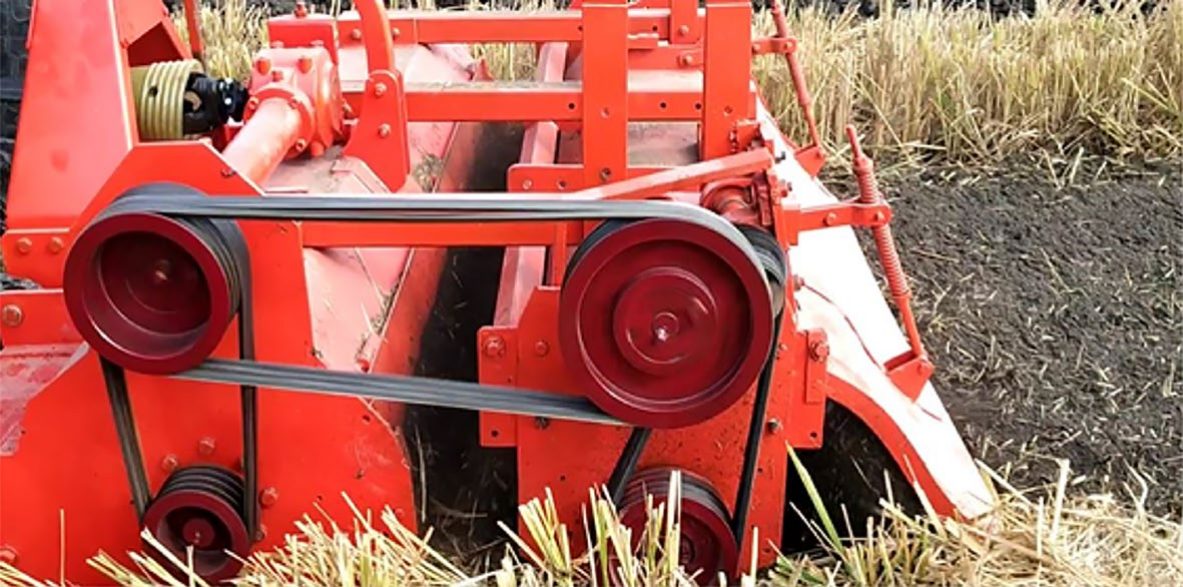
The developed residue management machine during the field test.

The field test was conducted at Norman Borlaug Crop Research Centre of Govind Ballabh Pant University of Agriculture and Technology Pantnagar, Uttarakhand, India. Located at 29°N latitude, 79.29°E longitude, and with an altitude of 243.80 m from the mean sea level in the Tarai belt of our country, as shown in Fig. 6. The tractor was used for field trials of 56 kW. The Moisture content (%), bulk density (kg/cm^3^), and soil strength (kg/cm^2^) of the test field are 13.63–14.30, 1.5–1.6, and 1.52, respectively. The independent and dependent parameters considered for performance evaluation of the developed residue management machine at field evaluation were forward speed (kmph), vertical adjustment between the rotor shaft of the straw chopper and rotavator (cm), rotary speed of straw chopper (rpm), and horizontal adjustment of rotor shaft of the straw chopper and rotavator (cm), field capacity (ha h^–1^), paddy residue size reduction (%), field efficiency (%), shredding efficiency (%), incorporation efficiency (%) and fuel consumption (l/h). The independent parameters of various levels are shown in Table 5. The trials were carried out after harvesting of paddy field by a combined harvester.

**Table 5:** Independent parameters and their levels, along with dependent parameters.

Independent Parameters meters	Levels	Dependent parameters
Forward speed (kmph)	Two levels—F_1_ & F_2_ (2.1 and 3.0)	Shredding efficiency (%) Incorporation efficiency (%) Size reduction of residue (%)
Vertical adjustment of cut (mm)	Two levels—V_1_ and V_2_ (100 and 200)	
Rotary speed of straw chopper (rpm)	Two levels—N_1_ and N_2_ (900 and 1100)	
Horizontal adjustment (mm)	Two levels (550 and 650)	

The selection of vertical and horizontal clearance are the key parameters that influence the structure and the operational performance of the developed machine. Excessive horizontal clearance will expand the machine frame, overloading the mounting linkage of the tractor. A too-small horizontal clearance will cause mutual interference between the two rotors, increasing the chance of stalk winding and blockage. The horizontal clearance should be optimal to ensure the machine's optimal performance. The horizontal space between the center of the straw chopper and rotavator rotor was kept at 550 mm, and 650 mm was kept under the study. The vertical space between the center of the straw chopper rotor and the rotavator rotor was kept at 100 mm and 200 mm^33^. The recommended rotary speed of the rotavator was kept at 180–210 rpm for proper pulverization of soil during incorporation of the paddy residue^30^; past researchers showed that the flail-type straw chopper performance was best at the rotary speed ranges between 750 to 1900 rpm^27^. The shape of rotavator blades for paddy residue incorporation generally used L type. Therefore L-type blades were considered for this study^32^. Inverted gamma-type blades work more satisfactorily than J-type and cutter bar serrated type blades^34^. Therefore, we have used inverted gamma-type blades in the straw chopper.

#### Statistical analysis

The total area utilized to evaluate the developed machine was around 2.5 ha. The area of the individual experimental plot size was about 30 × 15 m^2^. The total number of experiments was 16 and replicated these experiments in three times. The split-plot (4 × 4 × 3) design was used for the statistical evaluation of the straw chopper cum incorporation machine to analyze the effect of four independent variables, *i.e.,* the forward speed of the developed machine, rotor speed of the straw chopper, horizontal and vertical adjustments between the straw chopper and rotavator on the dependent variables like incorporation efficiency, paddy residue size reduction, and shredding efficiency. Analysis was done at a 5% level of significance. The experimental layout is shown in Table 6.

Abbreviations of arrangements were used under study:

- F_1_R_1_: Forward speed at a low level, i.e., 2.1 kmph, and rotary speed at a low level, i.e., 900 rpm.
- F_1_R_2_: Forward speed at a low level, i.e., 2.1 kmph, and rotary speed at a high level, i.e., 1100 rpm.
- F_2_R_1_: Forward speed at a high level, i.e., 3.0 kmph, and rotary speed at a low level, i.e., 900 rpm
- F_2_R_2_: Forward speed at a high level, i.e., 3.0 kmph, and rotary speed at a high level, i.e., 1100 rpm.
- H_1_V_1_: Horizontal adjustment at a low level, i.e., 550 mm, vertical adjustment at a low level, i.e., 100 mm.
- H_1_V_2_: Horizontal adjustment at a low level, i.e., 550 mm, vertical adjustment at a high level, i.e., 200 mm.
- H_2_V_1_: Horizontal adjustment at a high level, i.e., 650 mm, vertical adjustment at a low level, i.e., 100 mm.
- H_2_V_2_: Horizontal adjustment at a high level, i.e., 650 mm, vertical adjustment at a high level, i.e., 200 mm.

**Table 6:** Experimental layout for evaluation of paddy-developed residue management machine.

Replication 1				Replication 2				Replication 3			
Main Plot				Main Plot				Main Plot			
V_1_R_1_	V_1_R_2_	V_2_R_1_	V_2_R_2_	V_1_R_1_	V_1_R_2_	V_2_R_1_	V_2_R_2_	V_1_R_1_	V_1_R_2_	V_2_R_1_	V_2_R_2_

Sub Plot				Sub Plot				Sub Plot			
F_1_R_1_	F_1_R_1_	F_1_R_1_	F_1_R_1_	F_1_R_1_	F_1_R_1_	F_1_R_1_	F_1_R_1_	F_1_R_1_	F_1_R_1_	F_1_R_1_	F_1_R_1_
F_1_R_2_	F_1_R_2_	F_1_R_2_	F_1_R_2_	F_1_R_2_	F_1_R_2_	F_1_R_2_	F_1_R_2_	F_1_R_2_	F_1_R_2_	F_1_R_2_	F_1_R_2_
F_2_R_1_	F_2_R_1_	F_2_R_1_	F_2_R_1_	F_2_R_1_	F_2_R_1_	F_2_R_1_	F_2_R_1_	F_2_R_1_	F_2_R_1_	F_2_R_1_	F_2_R_1_
F_2_R_2_	F_2_R_2_	F_2_R_2_	F_2_R_2_	F_2_R_2_	F_2_R_2_	F_2_R_2_	F_2_R_2_	F_2_R_2_	F_2_R_2_	F_2_R_2_	F_2_R_2_

#### Soil and crop parameters.

The soil and crop parameters were measured before conducting the developed machine's field test and are shown in Tables 7 and 8.

**Table 7:** Soil parameters of a combined harvested field.

Sr. No	Particular	Range
1	The moisture content of soil (%)	13.63–14.30
2	Bulk density of soil (kg/cm^3^)	1.5–1.6
3	Soil strength (kg/cm^2^)	1.52

**Table 8:** Crop parameters of paddy residue field.

Sr. No	Particular	Range
1	The moisture content of residue (%)	22–30
2	Residue length (mm)	350–400
3	Residue available (t/ha)	8–9.5
4	Row-to-row spacing (mm)	22
5	Plant-to-plant spacing (mm)	15

*Machine parameters.*

The field experiment was conducted in a combined harvested paddy field to determine the developed residue management machine's fuel consumption, actual field capacity, theoretical field capacity, and field efficiency. The results of these studies are presented in Table 7. The time consumed for the individual experiment was about 7 to 8 min. The top-up method was used to measure fuel consumption during the study. The lowest fuel consumption of 12.5 l/h was observed when the rotary speed of the straw chopper and incorporator was at a low level, i.e., 900 rpm and 180 rpm, respectively (Table 7). The maximum actual and theoretical field capacities were about 0.35 and 0.64 ha/h, respectively (Table 7). The maximum field efficiency was observed at 60.46% Table 9.

**Table 9:** The field test included fuel consumption, efficiency, and actual and theoretical field capacity.

Sr. No	Parameter	Range
1	Fuel consumption (l/h)	12.5–14
2	Theoretical field capacity (ha/h)	0.43–0.64
3	Actual field capacity (ha/h)	0.26–0.35
4	Field efficiency (%)	55–60.46

#### Rotary speed shaft of the straw chopper

The speed of the rotor shaft of a straw chopper varies with the changing of gearbox gear and with the accelerator position. The use of a tachometer measured this rotary speed. A non-contacting type tachometer was considered in this study. Tags were pasted on the rotor shafts of the rotavator and straw chopper. The laser beam of the tachometer was focused on the tag, and the reading was shown on display in rpm.

#### Shredding efficiency

The shredding efficiency is the percentage of chopped straw paddy residue on the field after the operation concerning the straw paddy residue on the field operation.

14 $$E_{c} = \frac{F}{C} \times 100$$

where, E_c_ = Shredding efficiency of the machine, %; F = Amount of chopped paddy residue on the field after the operation, q/ha; C = Amount of paddy residue on the field before the operation, q/ha.

#### Paddy residue size reduction

It is the ratio of the average length of paddy residue after operation to the length before operation. For measuring the length of chopped paddy stubbles, approximately 100 g of chopped straw samples were collected in a polythene bag at each plot. Tags were kept for each sample for identification. To quantify chopped paddy residue, the sample was differentiated for size by measuring straw length manually with the help of a measuring scale. The paddy residue size reduction was determined by using the following relationship:

15 $$E_{b} = \frac{F}{B} \times 100$$

where, E_b_ = Paddy residue size reduction, %; F = Length of paddy residue after operation, mm; B = Length of paddy residue before operation, mm.

#### Incorporation efficiency

The incorporation efficiency was measured by the weight of paddy straw incorporated by the machine in the area of one square meter and weight after the operation of the developed residue management machine of paddy residue before the operation at the same location of the experimental plot. A percentage indicates it.

## Results

### Interaction among different types of speeds, vertical and horizontal adjustment on incorporation efficiency

The combined effect of rotary and forward speeds and vertical & horizontal adjustments have been presented in Table 10. Statistical analysis presented in Table 11 indicated that the effect of forwarding & rotary speeds, along with vertical & horizontal adjustments, were significant on incorporation efficiency. The maximum incorporation efficiency of paddy residue was obtained at arrangement V_1_H_2_F_1_R_2_ (95.31%), followed by V_1_H_1_F_I_R_2_ (94.30%). In contrast, the lowest incorporation efficiency was recorded at the arrangement V_1_H_2_F_2_R_2_ (59.42%) and V_1_H_1_F_2_R_2_ (59.43%), followed by V_2_H_1_F_2_R_2_ (64.63%) and V_2_H_2_F_2_R_2_ (64.98%). The value of the coefficient of difference was calculated, *i.e.,* 7.58. The incorporation efficiency for all arrangements in different combinations varied from 59.42% to 95.30%. The results showed that the incorporation efficiency was higher for rotary speed at the 2^nd^ level (1100 rpm), and incorporation efficiency was lower for forwarding speed at the 2^nd^ level (3.0 kmph). No statistical value difference is observed at various levels of horizontal arrangements on incorporation efficiency. A similar result showed that the percentage of burial residue increased with the average value of rotary speed. This is due to decreased bite cut or soil cutting frequency when rotary speed increases and forward speed decreases^36^. A similar pattern has been reported by Destain and Houmy^37^, who studied the rotary tiller's effect on soil structure.

**Table 10:** Effect of both speed and vertical adjustment and horizontal adjustment on incorporation efficiency.

Sr. No	Parameter	Incorporation efficiency, %
1	V_1_H_1_F_1_R_1_	85.26
2	V_1_H_1_F_1_R_2_	**94.30**
3	V_1_H_1_F_2_R_1_	79.93
4	V_1_H_1_F_2_R_2_	**59.43**
5	V_1_H_2_F_1_R_1_	85.44
6	V_1_H_2_F_1_R_2_	**95.31**
7	V_1_H_2_F_2_R_1_	79.93
8	V_1_H_2_F_2_R_2_	**59.42**
9	V_2_H_1_F_1_R_1_	81.33
10	V_2_H_1_F_1_R_2_	83.58
11	V_2_H_1_F_2_R_1_	78.79
12	V_2_H_1_F_2_R_2_	64.63
13	V_2_H_2_F_1_R_1_	81.09
14	V_2_H_2_F_1_R_2_	83.56
15	V_2_H_2_F_2_R_1_	78.42
16	V_2_H_2_F_2_R_2_	64.98
	SEm ±	2.60
	CD (*p* = 0.05)	7.58

(F_1_ = 2.1 kmph, F_2_ = 3.0 kmph_,_ R_1_ = 900 rpm and R_2_ = 1100 rpm); (V_1_ = 100 mm, V_2_ = 200 mm_,_ H_1_ = 550 mm and H_2_ = 650 mm).

**Table 11:** Analysis of variance for effect of operating parameters on incorporation efficiency.

Source of Variation	DF	Sum of squares	Mean squares	F-Calculated	Significance
Replication	2	47.301			
Factor A	3	96.845	32.282	7.261	0.02016
Error (a)	6	26.674	4.446		
Factor B	3	4,873.456	1,624.485	80.344	0.00000
Interaction A X B	9	427.955	47.551	2.352	0.04571
Error (b)	24	485.259	20.219		
Total	47	5,957.491			

Factors A and B combine horizontal & vertical clearance and forward speed, respectively.

### Interaction among different types of speeds, vertical and horizontal adjustment on size reduction of residue

The combined effect of rotary & forward speeds and horizontal & vertical adjustments on residue size reduction are shown in Table 12. Statistical analysis presented in Table 13 indicated that the effect of rotary, forward speed, and vertical and horizontal adjustment of the residue management machine was significant in the size reduction of a chopped paddy residue. The maximum size reduction of paddy residue was obtained for arrangements V_1_H_2_F_1_R_2_ (61.92%) followed by V_1_H_1_F_I_R_2_ (60.35%), V_1_H_1_F_1_R_1_ (54.76), and V_1_H_2_F_1_R_1_ (54.49%). The minimum size reduction was found for the arrangements V_2_H_2_F_2_R_2_ (22.99%) and V_2_H_1_F_2_R_2_ (24%), followed by V_2_H_1_F_2_R_1_ (24.13%). The statistical analysis revealed no significant difference in reduction in residue size for horizontal and vertical adjustment with a rotary and forward speed combination. The residue size reduction increased with an increase in rotary speed, whereas it decreased with an increase in forwarding speed. The results showed that when the forward speed was at a lower level (2.1 kmph), the higher size reduction of residue. The decrease in forwarding speed of the developed machine results that a lesser number of cuts per unit time of paddy residue, which results in a decrease in the percentage of height reduction of chopped paddy residue^38^.

**Table 12:** The interaction effect of speed, vertical, and horizontal adjustment on residue size reduction.

Sr. No	Parameter	Residue size reduction, %
1	V_1_H_1_F_1_R_1_	54.76
2	V_1_H_1_F_1_R_2_	60.35
3	V_1_H_1_F_2_R_1_	32.94
4	V_1_H_1_F_2_R_2_	33.00
5	V_1_H_2_F_1_R_1_	54.49
6	V_1_H_2_F_1_R_2_	61.92
7	V_1_H_2_F_2_R_1_	27.75
8	V_1_H_2_F_2_R_2_	27.49
9	V_2_H_1_F_1_R_1_	25.01
10	V_2_H_1_F_1_R_2_	31.88
11	V_2_H_1_F_2_R_1_	28.17
12	V_2_H_1_F_2_R_2_	24.00
13	V_2_H_2_F_1_R_1_	25.10
14	V_2_H_2_F_1_R_2_	31.75
15	V_2_H_2_F_2_R_1_	24.13
16	V_2_H_2_F_2_R_2_	22.99
	SEm ±	4.90
	CD (*p* = 0.05)	14.31

(F_1_ = 2.1 kmph, F_2_ = 3.0 kmph_,_ R_1_ = 900 rpm and R_2_ = 1100 rpm); (V_1_ = 100 mm, V_2_ = 200 mm_,_ H_1_ = 550 mm and H_2_ = 650 mm).

**Table 13:** Analysis of variance for effect of operating parameters on shredding efficiency.

Source of variation	DF	Sum of squares	Mean squares	F-Calculated	Significance
Replication	2	47.323			
Factor A	3	96.854	32.285	7.275	0.02007
Error (a)	6	26.625	4.437		
Factor B	3	4,873.455	1,624.485	80.339	0.00000
Interaction A X B	9	427.941	47.549	2.352	0.04572
Error (b)	24	485.287	20.220		
Total	47	5,957.485			

Factors A and B combine horizontal & vertical clearance and forward speed, respectively.

### Interaction among different types of speeds, vertical and horizontal adjustment on shredding efficiency

Statistical analysis presented in Table 14 indicated that different forward and rotary speeds and vertical and horizontal adjustments were significant in shredding efficiency. The shredding efficiency of the residue management machine varied between 4.69% and 40.57% for all the combinations of arrangements, as shown in Table 15. The minimum shredding efficiency of paddy residue was achieved for arrangements V_1_H_2_F_1_R_2_ (4.69%), followed by V_1_H_1_F_I_R_2_ (5.70%). The maximum shredding efficiency was observed for V_1_H_2_F_2_R_2_ (40.58%) and V_1_H_1_F_2_R_2_ (40.57%). It was observed that the shredding efficiency was higher when the forward speed was 3.0 kmph. The lowest shredding efficiency was found at the forward speed at a lower level (2.1 kmph). The effect of various levels of horizontal arrangements and the same levels of forwarding and rotary speed on shredding efficiency was not found significant.

**Table 14:** Analysis of variance for effect of operating parameters on paddy residue size reduction.

Source of variation	DF	Sum of squares	Mean squares	F-Calculated	Significance
Replication	2	161.348			
Factor A	3	3,328.009	1,109.336	163.940	0.00000
Error (a)	6	40.600	6.767		
Factor B	3	1,878.848	626.283	9.912	0.00019
Interaction A X B	9	122.644	13.627	0.216	0.98928
Error (b)	24	1,516.394	63.183		
Total	47	7,047.843			

Factors A and B combine horizontal & vertical clearance and forward speed, respectively.

**Table 15:** Effect of both speed and vertical adjustment and horizontal adjustment on shredding efficiency.

Sr. No	Arrangement	Shredding efficiency, %
1	V_1_H_1_F_1_R_1_	14.74
2	V_1_H_1_F_1_R_2_	5.70
3	V_1_H_1_F_2_R_1_	20.07
4	V_1_H_1_F_2_R_2_	40.57
5	V_1_H_2_F_1_R_1_	14.56
6	V_1_H_2_F_1_R_2_	4.69
7	V_1_H_2_F_2_R_1_	20.07
8	V_1_H_2_F_2_R_2_	40.58
9	V_2_H_1_F_1_R_1_	18.67
10	V_2_H_1_F_1_R_2_	16.42
11	V_2_H_1_F_2_R_1_	21.21
12	V_2_H_1_F_2_R_2_	35.37
13	V_2_H_2_F_1_R_1_	18.91
14	V_2_H_2_F_1_R_2_	16.44
15	V_2_H_2_F_2_R_1_	21.58
16	V_2_H_2_F_2_R_2_	35.02
	SEm ±	2.60
	CD (*p* = 0.05)	7.58

(F_1_ = 2.1 kmph, F_2_ = 3.0 kmph_,_ R_1_ = 900 rpm and R_2_ = 1100 rpm); (V_1_ = 100 mm, V_2_ = 200 mm_,_ H_1_ = 550 mm and H_2_ = 650 mm).

Consequently, the results show a decrease in the chopped length of the straw as the machine's forward speed is increased. It may be that the decrease in the chopped length of the straw is a result of increasing forward speed, which will reduce slippage and ensure proper compression of the straw because there is more straw to be cut, i.e., feed rate will increase as forward speed increases. In addition, during the experiment, it was observed that the chopped length of the straw decreased as the speed of the cylinder increased from 1600 to 1800 revolutions per minute. During low-speed operation, the blades of the chopping cylinder move faster relative to the material than they do during high-speed operation, which results in a maximum cut of straw, which results in a minimum chopped length of the straw, and this is caused by several cuts per unit time and high speeds^36,39^.

## Discussion

The results show that the incorporation efficiency is proportional to rotary speed and inversely proportional to forwarding speed. As the rotary speed of developed residue management machines increases, the number of cuts per unit of soil and paddy residue increases, leading to increased incorporation of paddy residue and vice-versa. This trend of results was similar to the study conducted by Satish^40^. The results showed that when the forward speed was at a lower level (2.1 kmph), the higher size reduction of residue. The decrease in forwarding speed of the developed machine resulted in fewer cuts per unit time of paddy residue, which resulted in a decrease in the percentage of height reduction of chopped paddy residue and was due to less time available for cutting paddy residue at a higher forward speed^38^. The trend shows that the shredding efficiency was higher when the forward speed was 3.0 kmph. In contrast, the lowest shredding efficiency was found at the forward speed at a lower level (2.1 kmph). The forward speed of the developed residue management machine increased the number of cuts per unit time per unit length decrease, decreasing the percentage weight of chopped paddy residue, leading to decreased shredding efficiency^38^. Rather than develop a crop residue machine, Sidhu et al. (2015)^34^ develop and evaluate Turbo Happy Seeder for sowing wheat into heavy rice residues in NW India. Several on-farm trials have demonstrated that 9-row Turbo Happy Seeders are equal or superior to conventional tillage and straw burning for wheat sowing within rice residues. Zhang et al. (2017)^1^ summarized the global research on blade design, blade arrangement, and power consumption based on two main key factors of the quality of residue chopping and spreading uniformity. Dai et al. (2010)^41^ developed a straw chopper with a fast-decomposing inoculant sprayer to speed up the decomposition of straw mulch on the soil. A straw chopper and decomposing inoculant sprayer combine to make the machine. Before the straw chopper chops the crop straw, the decomposing inoculant sprayer sprays the decomposing inoculant (microbial inoculant) into the air. It runs on a 22.4 kW engine and chops straw into 5 to 9-cm pieces.

While the machine has many advantages over other machinery, it is still difficult for many farmers to adopt this seeder due to significant challenges. One of the main barriers to adopting the machine is its high capital cost, the risk aversion on the part of farmers (particularly those from marginal or small farms), and heavy subsidies on electricity and diesel for agriculture. During the awareness-raising phase, subsidies for machinery have played a pivotal role in making the technology more appealing. In many areas, farmers still prefer clean fields with low residue levels. For farmers, burning rice residues is an easy and inexpensive way to clear their fields. Several field demonstrations with the crop residue management machine need to be conducted on various soil types under different climates and seasons to overcome these problems.

## Conclusions

Despite the extensive research on straw chopper structure/residual management and operating parameters, crop straw mechanical, physical properties, and machine throughput that has been conducted to improve crop straw chopping quality, very few attempts have been made to relate these parameters to cutting and spreading. The following major conclusions can be drawn from this study:

- The actual trials of the developed residue management machine were found satisfactory.
- The developed residue management machine's theoretical field capacity, effective capacity, and field efficiency were 0.43–0.64 ha/h, 0.26–0.35 ha/h, and 55–60.46%, respectively.
- The fuel consumption of the developed machine was recorded at 12.5–14lph.
- The incorporation efficiency of paddy residue by straw chopper cum incorporator ranges between 59.42% and 95.31%.
- The trash size reduction of paddy reissue by straw chopper cum incorporator lies in the range of 22.99–24.13%.
- The maximum shredding efficiency was recorded at about 40.58%.

Despite the novelty and practical contribution of the development presented in this study, the main limitation is that it requires a high-power range tractor (> 60 hp), which was based on the Indian farmers' suggestions that the power transmission system could modify. The belt and pulley transmission is used in the developed residue management machine. It observed that more heat is generated between the belt and pulley whenever the load increases at the incorporator. Therefore, this power transmission system does not work for longer. The power transmission used in straw chopper cum incorporator cannot run for a long period.

Nevertheless, the machine's weight can be reduced by making a single frame for both the chopping unit and incorporation unit; hence the power requirement of the residue management machine can be reduced further.

## Data and materials availability

The datasets used and/or analyzed during the current study are available from the corresponding author on reasonable request.

## References

[1] Zhang Z (2017). Global overview of research and development of crop residue management machinery. Appl. Eng. Agric..

[2] Lohan SK (2018). Burning issues of paddy residue management in north-west states of India. Renew. Sustain. Energy Rev..

[3] Jin H, Qingjie W, Hongwen L, Lijin L, Huanwen G (2009). Effect of alternative tillage and residue cover on yield and water use efficiency in annual double cropping system in North China plain. Soil Tillage Res..

[4] Anonymous. *India Agri Stat. Year wise production under food grain crops in India 2016–2017. Agricultural Statistics at a Glance: Ministry of Agriculture & Farmers Welfare*. https://www.indiastatagri.com/ (2019).

[5] Monforti F, Bódis K, Scarlat N, Dallemand J-F (2013). The possible contribution of agricultural crop residues to renewable energy targets in Europe: A spatially explicit study. Renew. Sustain. Energy Rev..

[6] Bhattacharyya SC (2014). Viability of off-grid electricity supply using rice husk: A case study from South Asia. Biomass Bioenerg..

[7] Gupta, R. *Causes of emissions from agricultural residue burning in north-west India: Evaluation of a technology policy response*. (2012).

[8] Hayashi K (2014). Trace gas and particle emissions from open burning of three cereal crop residues: Increase in residue moistness enhances emissions of carbon monoxide, methane, and particulate organic carbon. Atmos. Environ..

[9] Koopmans, A. & Koppejan, J. Agricultural and forest residues-generation, utilization and availability. In *Regional consultation on modern applications of biomass energy* vol. 6 10 (Kuala Lumpur, Malaysia, 1997).

[10] Singh J (2018). Paddy and wheat stubble blazing in Haryana and Punjab states of India: A menace for environmental health. Environ. Qual. Manag..

[11] Singh R, Yadav DB, Ravisankar N, Yadav A, Singh H (2020). Crop residue management in rice–wheat cropping system for resource conservation and environmental protection in north-western India. Environ. Dev. Sustain..

[12] Gupta PK (2004). Residue burning in rice–wheat cropping system: Causes and implications. Curr. Sci..

[13] NABARD. *Sectoral Paper on Farm Mechanization: Farm Sector Policy Department, NABARD Head Office, Bandra East, Mumbai, Maharashtra 400051*. https://www.nabard.org/ (2018).

[14] Singh S (2002). Annual report of project Mechanization of rice-wheat cropping systems for increasing the productivity 2001–02.

[15] Bimbraw AS (2019). Generation and impact of crop residue and its management. Curr. Agric. Res. J..

[16] Devi S, Gupta C, Jat SL, Parmar MS (2017). Crop residue recycling for economic and environmental sustainability: The case of India. Open Agric..

[17] Hobbs, P. R. & Morris, M. L. Meetings South Asia’s future food requirements from rice–wheat cropping systems; priorities issues facing researchers in the post green revolution era. NRG Paper 96–101 (2002).

[18] NPMCR. National Policy for Management of Crop Residues (NPMCR). *Government of India Ministry of Agriculture Department of Agriculture & Cooperation (Natural Resource Management Division) Krishi Bhawan, New Delhi* http://agricoop.nic.in/sites/default/files/NPMCR_1.pdf (2019).

[19] Kanokkanjana K, Garivait S (2013). Alternative rice straw management practices to reduce field open burning in Thailand. Int. J. Environ. Sci. Dev..

[20] Børresen T (1999). The effect of straw management and reduced tillage on soil properties and crop yields of spring-sown cereals on two loam soils in Norway. Soil Tillage Res..

[21] Wei T (2015). Effects of wheat straw incorporation on the availability of soil nutrients and enzyme activities in semiarid areas. PLoS ONE.

[22] Singh B, Shan YH, Johnson-Beebout SE, Singh Y, Buresh RJ (2008). Crop residue management for lowland rice-based cropping systems in Asia. Adv. Agron..

[23] Singh Y, Singh B, Timsina J (2005). Crop residue management for nutrient cycling and improving soil productivity in rice-based cropping systems in the tropics. Adv. Agron..

[24] Amran MA (2021). Value-added metabolites from agricultural waste and application of green extraction techniques. Sustainability.

[25] Rehman A, Ullah A, Nadeem F, Farooq M, Farooq M, Pisante M (2019). Sustainable Nutrient Management. Innovations in Sustainable Agriculture.

[26] Sah G (2015). Effects of tillage and crop establishment methods, crop residues, and nitrogen levels on wheat productivity, energy-savings and greenhouse gas emission under rice -wheat cropping system. Nepal J. Sci. Technol..

[27] Garg IK (2004). Design and development of rice straw chopper-cum-spreader. J. Res..

[28] Garg, I. K. Design, development and evaluation of Roto till drill. In *Souvenir of 36th annual convention of ISAE, Indian Institute of Technology, Kharagpur, India, *January 28–30th 9 (2002).

[29] Sidhu, H. S., Singh, M., Blackwell, J., Bector, V. & Singh, M. Development of the Combo Happy Seeder for Direct Drilling into a Combine Harvested Paddy Field. In *39th Annual Convention & Symposium of the Indian Society of Agricultural Engineers, *4 February 2004 (2005).

[30] Sidhu, H. S., Goyal, R., Sharda, A. & Singh, M. Design and Development of Straw Manager cum Spreader for Combine Harvester. In *A paper presented at 41st annual convention and symposium of Indian Society of Agricultural engineering. *Junagadh Agricultural University, Junagadh, Gujrat, India (2006).

[31] Ramulu C, Pateriya RN, Deepshika A, Naik MA (2018). Machinery for residue management of different crops: A review. J. Pharmacogn. Phytochem..

[32] Sharda A, Singh S (2004). Effect of selected parameters on field performance of rotary tiller. J. Inst. Eng..

[33] Jia H, Wang L, Li C, Tan H, Ma C (2010). Combined stalk–stubble breaking and mulching machine. Soil Tillage Res..

[34] Sidhu HS (2015). Development and evaluation of the turbo happy seeder for sowing wheat into heavy rice residues in NW India. F. Crop. Res..

[35] Khurmi RS, Gupta JK (2005). A textbook of machine design.

[36] Pateriya RN (2021). Studies on performance of a rotavator as affected by its λ-ratio. Int. J. Curr. Microbiol. Appl. Sci..

[37] Destain M-F, Houmy K (1990). Effects of design and kinematic parameters of rotary cultivators on soil structure. Soil Tillage Res..

[38] Virk, G. Performance evaluation of paddy straw chopper with combine cutting head mechanism: M. Tech Thesis. (Punjab Agricultural University, Ludhiana, India, 2016).

[39] Singh A, Dhaliwal IS, Dixit A (2011). Performance evaluation of tractor mounted straw chopper cum spreader for paddy straw management. Indian J. Agric. Res..

[40] Satish, D. A. Refinement and evaluation of straw bruising and sieving system for wheat straw combine: M. Tech Thesis. (Punjab Agricultural University, Ludhiana, India, 2014).

[41] Dai F (2010). Design and experiment on straw returning machine with fast decomposing inoculant spray equipment. Nongye Jixie Xuebao Trans Chinese Soc. Agric. Mach..

